# Intraventricular hemorrhage in asphyxiated newborns treated with hypothermia: a look into incidence, timing and risk factors

**DOI:** 10.1186/s12887-015-0415-7

**Published:** 2015-08-28

**Authors:** Ghalia Al Yazidi, Elodie Boudes, Xianming Tan, Christine Saint-Martin, Michael Shevell, Pia Wintermark

**Affiliations:** Division of Pediatric Neurology, Department of Pediatrics, Montreal Children’s Hospital, McGill University, Montreal, Canada; Division of Newborn Medicine, Department of Pediatrics, Montreal Children’s Hospital, McGill University, 1001 Boul. Decarie, Site Glen, Block E, EM0.3244, Montreal, H4A 3J1 QC Canada; Biostatistics Core Facility, Research Institute, McGill University Health Centre, Montreal, Canada; Department of Radiology, Montreal Children’s Hospital, McGill University, Montreal, Canada

**Keywords:** Birth asphyxia, Hypothermia, Neonatal encephalopathy, Intraventricular hemorrhage, Magnetic resonance imaging, Newborn brain

## Abstract

**Background:**

Intraventricular hemorrhage (IVH) is uncommon in term newborns. Asphyxia and hypothermia have been mentioned separately as possible risk factors of IVH, since they might cause fluctuations of cerebral blood flow. The aim of this study was to assess the incidence, the timing, and the risk factors of intraventricular hemorrhage (IVH) in term asphyxiated newborns treated with hypothermia.

**Methods:**

We conducted a prospective cohort study of all term asphyxiated newborns treated with hypothermia from August 2008 to June 2013. The presence or not of IVH was assessed using brain magnetic resonance imaging (MRI) performed after the hypothermia treatment was completed or using head ultrasound during the hypothermia treatment. For these newborns, to determine the timing of IVH, we retrospectively reviewed if they had other brain imaging studies performed during their neonatal hospitalization stay. In addition, we compared their general characteristics with those not developing IVH.

**Results:**

One hundred and sixty asphyxiated newborns met the criteria for hypothermia. Fifteen of these newborns developed IVH, leading to an estimate of 9 % (95 % CI: 5.3-15.0 %) of IVH in this population of newborns. Fifty-three percent had hemorrhage limited to the choroid plexus or IVH without ventricular dilatation; 47 % had IVH with ventricular dilatation or parenchymal hemorrhage. Sixty-seven percent had an initial normal brain imaging; the diagnostic brain imaging that demonstrated the IVH was obtained either during cooling (in 30 %), within 24 h of the rewarming (in 30 %), or 24 h after the rewarming (in 40 %). Recurrent seizures were the presenting symptom of IVH during the rewarming in 20 % of the newborns. Coagulopathy was more frequent in the asphyxiated newborns developing IVH (*p < 0.001*). The asphyxiated newborns developing IVH also presented more frequently with persistent pulmonary hypertension, hypotension, thrombocytopenia and coagulopathy (*p = 0.03*).

**Conclusions:**

The asphyxiated newborns treated with hypothermia appear to be at an increased risk of IVH, especially those with significant hemodynamic instability. IVH seems to develop during late hypothermia and rewarming. Efforts should be directed towards maintaining hemodynamic stability in these patients, even during the rewarming.

## Background

Intraventricular hemorrhage is uncommon in term newborns [1–3]. Asphyxia and hypothermia have been mentioned separately as possible risk factors of IVH, since they might cause fluctuations of cerebral blood flow [3–5]. Neonatal encephalopathy in term newborns remain a serious condition responsible for significant mortality (*i.e.*, 23 % of all neonatal deaths worldwide or an estimated 900,000 deaths each year) [6–8] and long-term morbidity, including cerebral palsy and intellectual disability. At present, therapeutic hypothermia is the only available neuroprotective treatment for these newborns [9], with an improved survival and neurological outcome at 6 years [10]. The IVH that develops in the context of neonatal encephalopathy may cause an additional barrier for the optimal development of these newborns. Studies are needed to understand whether asphyxiated newborns treated with hypothermia are at a greater risk of developing IVH, when they are the most at risk for it (*i.e.*, during hypothermia, during rewarming, or after the completion of treatment), and what are the risk factors for developing IVH. To our knowledge, the total body hypothermia (TOBY) study group is the only hypothermia trial study that has reported intracranial hemorrhage as an adverse outcome in the context of neonatal encephalopathy and therapeutic hypothermia, with a reported incidence of intracranial hemorrhages in the hypothermic newborns of 39 % versus 31 % in the normothermic newborns. However, intracranial hemorrhages included 85 % of subdural hematomas, and the exact frequency of IVH was not described [11].

We hypothesized that asphyxiated newborns may be at an increased risk of developing intraventricular hemorrhage when treated with therapeutic hypothermia. Thus, the objective of this study was to determine the incidence of intraventricular hemorrhage in term asphyxiated newborns treated with hypothermia and to identify the timing and risk factors associated with IVH.

## Methods

We conducted a prospective cohort study of term asphyxiated newborns admitted to our neonatal intensive care unit from August 2008 to June 2013, who met the following criteria for induced hypothermia [11–13]: (1) gestational age ≥ 36 weeks and birth weight ≥ 1800 g; (2) evidence of fetal distress, *i.e.*, history of an acute perinatal event, cord pH ≤ 7.0 or base deficit ≤ − 16 mEq/L; (3) evidence of neonatal distress, such as an Apgar score ≤ 5 at 10 min, postnatal blood gas pH obtained within the first hour of life ≤ 7.0 or base deficit ≤ − 16 mEq/L, or a continued need for ventilation initiated at birth and continued for at least 10 min; (4) evidence of moderate to severe neonatal encephalopathy by an abnormal neurological exam and/or amplitude-integrated electroencephalogram. Eligible patients received whole-body cooling to an esophageal temperature of 33.5 °C, initiated within the first 6 h of life, continued for 72 h, and then they were slowly rewarmed using standard protocol (*i.e.*, temperature increase of 0.5 °C per hour). The prospectively collected database, including clinical characteristics, imaging results and placenta results was approved by the Research Ethics Board of McGill University Health Centre, who waived the need for informed consent as data were collected from the charts without requiring any additional testing in the newborns.

As per the current standard protocol in our neonatal intensive care unit to evaluate for brain injury in these newborns, only a brain magnetic resonance imaging (MRI) was performed after the hypothermia treatment was completed, except for the very sick newborns who may die from the complications of neonatal encephalopathy for whom a head ultrasound was requested at the bedside during hypothermia treatment. The MRI scans were performed using a 3 T clinical system (Achieva X, Philips Healthcare, Best, The Netherlands). Each MRI study included a 3D T1-weighted gradient-echo (TR/TE, 24/4.6 ms; matrix size, 180 × 180; FOV, 180 mm; flip angle, 30 degree; 104 sagittal slices, with a section thickness of 1.0 mm and multiplanar reformations in axial and coronal planes), a turbo spin-echo (TSE) high resolution T2-weighted (TR/TE, 5000/90 ms; TSE factor, 15; matrix size, 300x300; FOV, 150 mm; flip angle, 90 degree; 27 axial sections, with a section thickness of 3.0 mm), a single shot echo-planar imaging (EPI) diffusion-weighted imaging sequence (DWI) (TR/TE, 2424.4/69 ms; matrix size, 200 × 117; FOV, 240 mm; b values 600 and 1200 s/mm^2^; flip angle, 90 degree; 21 axial sections, with a section thickness of 4.0 mm) and a gradient echo (GRE) T2*-weighted sequence (TR, shortest; TE, 16 ms; matrix size, 384 × 384; flip angle, 18 degree; 21 axial sections, with a section thickness of 4 mm). In addition, since 2010, when possible (*i.e.*, when the parents consented for their newborns to have additional MRIs if the newborns were hemodynamically stable and when a team of a nurse and a respiratory therapist was available to go to the MRI), newborns were enrolled in an MRI research study, and MRI scans were performed on day 1 of life, day 2–3 of life, around day 10 of life and/or around 1 month of life. These time-points were chosen to ensure that no antenatal brain injury was present (day 1 of life), to assess early patterns of injury (day 2–3 of life), and to define the extent of definitive brain injuries (around day 10 of life and around 1 month of life). Patients receiving hypothermia had their therapy maintained during the MRI scan without any adverse events [14]. Any ventilation, pressor support, or sedation was maintained during the MRI scanning process; additional sedation was avoided. A similar imaging protocol was used with these newborns at the different time-points. The head ultrasound scans were performed at the bedside in the neonatal intensive care unit according to standard protocols using a Toshiba Applio 2D multifrequency 4.2-9.0 MHz pediatric head probe. All these MRI scans and head ultrasounds were used to determine the incidence of IVH among this population of newborns. Neuroradiologists, who were blinded to the clinical condition of the newborns, interpreted the brain imaging studies of the asphyxiated newborns treated with hypothermia. They reported the severity of intraventricular hemorrhage, the presence and extent of brain injury as per the previously described magnetic resonance imaging scoring system [15], and other intracranial bleeding. *Intraventricular hemorrhage* was categorized as either a hemorrhage limited to the choroid plexus in the lateral ventricle, an IVH without ventricular dilatation, an IVH with ventricular dilatation, or a parenchymal hemorrhage. For the newborns with IVH, we then retrospectively reviewed if they had other brain imaging studies performed during their neonatal hospitalization stay, since the attending physician caring for the newborn may have decided to order additional imaging.

The general characteristics of the newborns included in our cohort were collected, including gestational age, birth weight, sex, mode of delivery, Apgar score at 10 min, arterial cord pH, initial blood gas pH, and highest serum lactate level. The presence of possible symptoms of IVH was recorded as well, including apneas, desaturations, bradycardia, seizures, and lethargy.

The described risk factors for intraventricular hemorrhage also were collected. The definition for these risk factors was adapted from the Cochrane database review of these patients [16] so we could compare our numbers with those in the literature. *Mechanical ventilation* was defined as the need for conventional ventilation or an oscillator for ventilation. *Persistent pulmonary hypertension (PPHN)* was diagnosed clinically when there was a persistent significant difference of ≥ 10 in pre-ductal and post-ductal oxygen saturation levels [16]; most often, an echocardiogram was also performed to confirm the suprasystemic pulmonary hypertension. *Hypotension* was defined as a mean arterial pressure < 40 mmHg [15]. *Thrombocytopenia* was defined as a platelet count < 50 x 109/L [16]. *Coagulopathy* was defined as a prolonged coagulation time or hemorrhage [16]. *Hypoglycaemia* was defined as glucose < 2.6 mmol/L on at least one occasion [16]. The combination of persistent pulmonary hypertension, hypotension, thrombocytopenia, and coagulopathy was considered in this manuscript as *hemodynamic instability*.

Statistical analysis was performed to assess the differences between the newborns developing or not developing IVH. Differences were also evaluated between the newborns with a hemorrhage limited to the choroid plexus vessels in the lateral ventricle or an IVH without ventricular dilatation and the newborns with an IVH with ventricular dilatation or parenchymal hemorrhage. Fisher’s exact tests were used for categorical data and Mann–Whitney U tests for continuous data*. A p* value *< 0.05* was determined as significant.

## Results

**Fig. 1:**
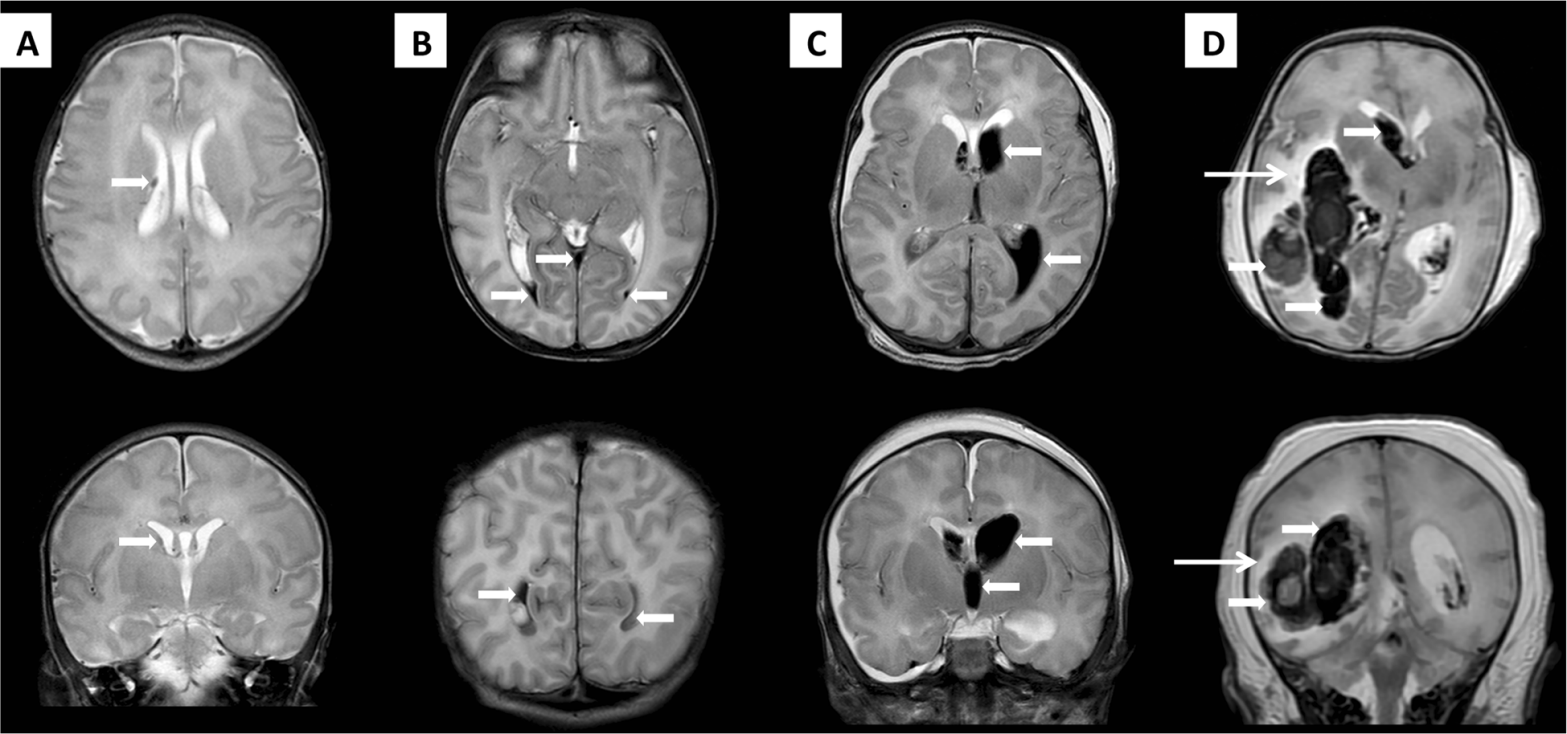
Brain magnetic resonance imaging in asphyxiated newborns treated with hypothermia who developed intraventricular hemorrhage, axial T2-weighted imaging (superior row) and coronal T2-weighted imaging (inferior row). **a** Brain magnetic resonance imaging in an asphyxiated newborn developing hemorrhage limited to the choroid plexus in the lateral ventricle (thick arrows). **b** Brain magnetic resonance imaging in an asphyxiated newborn developing an IVH without ventricular dilatation, showing the intraventricular hemorrhage in the lateral ventricles and the third ventricle (thick arrows) without dilatation of the ventricles. **c** Brain magnetic resonance imaging in an asphyxiated newborn developing IVH with ventricular dilatation, showing the intraventricular hemorrhage with dilatation of the lateral ventricles and the third ventricle (thick arrows). **d** Brain magnetic resonance imaging in an asphyxiated newborn developing IVH and parenchymal hemorrhage, showing a large right intraventricular and parieto-occipital parenchymal hemorrhage (thick arrows) with some extension into the left ventricle. Possible infarcted zones in the bilateral watershed areas were noted (thin arrow)

One hundred and sixty term asphyxiated newborns met the criteria for therapeutic hypothermia. Fifteen of these newborns developed documented intraventricular hemorrhage, leading to an estimate of 9 % (95 % CI: 5.3-15.0 %) of intraventricular and/or intraparenchymal hemorrhage in this population of newborns. Among them, 53 % (8/15) developed a hemorrhage limited to the choroid plexus in the lateral ventricle or an IVH without ventricular dilatation; 47 % (7/15) had an IVH with ventricular dilatation or a parenchymal hemorrhage (Fig. 1). Three of these patients (20 % [3/15]) died from the complications of neonatal encephalopathy: two died before the hypothermia treatment was completed, and one died at one week of life.

Among the fifteen asphyxiated newborns in our cohort who developed intraventricular hemorrhage, 67 % (10/15) had an initial brain imaging study that did not show any intraventricular hemorrhage. In these patients, the diagnostic brain imaging that permitted the demonstration of the intraventricular hemorrhage was obtained either during cooling in 30 % (3/10), within 24 h of the rewarming in 30 % (3/10), or 24 h after rewarming in 40 % (4/10) (Table 1).

**Table 1:** Timing of imaging and seizures in term asphyxiated newborns treated with hypothermia developing intraventricular hemorrhage (IVH)

Patient #	Before or during cooling Day 1 of life	During cooling Days 2–4 of life	During rewarming Day 4 of life	After cooling and rewarming Days 4–6 of life	After cooling and rewarming Days 7–15 of life	Brain injury^a^	Other intracranial bleeding
Term asphyxiated newborns treated with hypothermia and developing IVH (*n* = 15)							
Severity of IVH							
IVH limited to the choroid plexus vessels in the lateral ventricles or without ventricular dilatation (*n* = 8)							
1				HUS day 4 no IVH	MRI day 7 IVH	None	subdural hematomas
2	HUS day 1 no IVH				MRI day 10 IVH	CGM + WM	subdural hematomas
3		HUS day 3 no IVH			MRI day 10 IVH	None	subdural hematomas
4	HUS day 1 no IVH			MRI day 4 IVH	N/A	CGM + WM	None
5^b^	HUS day 1 no IVH	HUS day 2 IVH			N/A	CGM + WM	subdural hematomas
6				MRI day 5 IVH	N/A	None	None
7	MRI day 1 no IVH	MRI day 2 IVH			MRI day 10 IVH	None	subdural hematomas
8					MRI day 14 IVH	None	subdural hematomas
IVH with ventricular dilatation or parenchymal hemorrhage (*n* = 7)							
9^b^			seizure	HUS day 4 IVH	N/A	N/A	N/A
10		MRI day 3 no IVH	seizure		MRI day 13 IVH	CGM + WM + BG	subdural + epidural hematoma
11	MRI day 1 IVH	MRI day 2 IVH			MRI day 10 IVH	None	none
12	HUS day 1 no IVH			CT day 5 IVH	MRI day 10 IVH	CGM + WM	None
13		MRI day 3 IVH			MRI day 10 IVH	None	subdural hematomas
14^a^	HUS day 1 no IVH	HUS day 2 IVH			N/A	N/A	N/A
15		HUS day 2 no IVH	seizure	MRI day 5 IVH	MRI day 10 IVH	CGM+WM+BG	None

^a^Each MRI or autopsy was categorized as brain injury predominantly in the basal ganglia (BG), predominantly in the watershed areas (CGM + WM), or in the cortical grey matter, white matter and basal ganglia (CGM + WM + BG)

^b^These patients died from the complications of neonatal encephalopathy. Patient #5 did not have a brain MRI to define the extent of brain injury, but had an autopsy. Patients #9 and #14 did not have a brain MRI or an autopsy to define the extent of brain injury

*Abbreviations*: *CT* computed tomography, *HUS* head ultrasound, *MRI* magnetic resonance imaging, CGM cortical grey matter, WM white matter, BG basal ganglia

Recurrent seizures appeared to be the presenting symptoms of IVH in one of the asphyxiated newborns. Two other newborns - who had episodes of seizures in the first hours of life that initially were well controlled with a single anti-epileptic medication - developed refractory seizures coinciding with the time of the diagnosis of their IVH (Table 1). The recurrent seizures in all three patients presented during the rewarming. No other presenting symptoms such as apnea, desaturation, bradycardia, desaturation, or lethargy were recorded. A determination of the timing of the IVH was not possible in the remaining newborns, since they did not have an initial normal brain imaging performed before their diagnostic imaging, and/or they did not develop any symptoms.

The clinical characteristics of all the patients are shown in Table 2. The gestational age, birth weight, sex, Apgar score at 10 min, arterial cord pH, initial blood gas pH, and highest serum lactate level were similar between the asphyxiated newborns who developed and did not develop IVH. Also, these characteristics were not different according to the severity of the observed IVH. Males who developed IVH presented more often with IVH with ventricular dilatation or parenchymal hemorrhage rather than IVH limited to the choroid plexus vessels or IVH without ventricular dilatation (*p = 0.007*).

**Table 2:** Clinical characteristics of the study patients

Clinical characteristics of the study patients	General population of term asphyxiated newborns treated with hypothermia (*n* = 145)	Term asphyxiated newborns treated with hypothermia and developing IVH (*n* = 15)	*p* value	IVH limited to the choroid plexus vessels in the lateral ventricles or IVH without ventricular dilatation (*n* = 8)	IVH with ventricular dilatation or parenchymal hemorrhage (*n* = 7)	*p* value
Gestational age (weeks), mean ± SD	39.2 ± 1.5	39.1 ± 1.7	NS	39.5 ± 1.9	38.6 ± 1.3	NS
Birth weight (g), mean ± SD	3420 ± 652	3274 ± 659	NS	3422 ± 673	3091 ± 590	NS
Gender, n (%)			NS			0,007
Male, n (%)	78 (54)	9 (60)		2 (25)	7 (100)	
Female, n (%)	67 (46)	6 (40)		6 (75)	0 (0)	
Apgar score < 5 at 10 min, n (%)	74 (51)	8 (53)	NS	7 (88)	5(71)	NS
Arterial cord pH, mean ± SD	6,99 ± 0.20	7.03 ± 0.16	NS	7.00 ± 0.16	7.06 ± 0.16	NS
Initial postnatal blood gas pH, mean ± SD	7.03 ± 0.19	7.07 ± 0.16	NS	7.09 ± 0.12	7.06 ± 0.19	NS
Highest lactate level (mmol/L), mean ± SD	9.12 ± 5.67	10.02 ± 5.53	NS	9.36 ± 4.70	10.77 ± 6.66	NS

*Abbreviations*: *IVH* intraventricular hemorrhage, *NS* non-significant

The known risk factors of IVH were collected and are shown in Table 3. Sixty-seven percent (10/15) of the asphyxiated newborns developing IVH needed mechanical ventilation, with 46 % (6/13) still needing it during the rewarming period. Forty percent (6/15) presented with persistent pulmonary hypertension, with 38 % (5/13) requiring inhaled nitric oxide through the rewarming phase as well. Sixty-seven percent (10/15) presented hypotension; and 54 % (7/13) still required inotropic support during the rewarming period. Thrombocytopenia was present in 47 % (7/15) of the newborns, and coagulopathy was present in 73 % (11/15) of them; and 46 % (6/13) still presented significant thrombocytopenia and coagulopathy throughout the rewarming. Hypoglycemia was present in 20 % (3/15). Thirty-three percent (5/15) of the newborns had a history of an instrumental assisted delivery. All asphyxiated newborns developing IVH with ventricular dilatation or parenchymal hemorrhage who presented with significant hypotension, persistent pulmonary hypertension and coagulopathy during the hypothermia treatment still had these risk factors present during the rewarming period.

**Table 3:** Potential risk factors in term asphyxiated newborns treated with hypothermia and developing intraventricular hemorrhage

	General population of term asphyxiated newborns treated with hypothermia (*n* = 145)	Term asphyxiated newborns treated with hypothermia and developing IVH (*n* = 15)	*p* value	IVH limited to the choroid plexus vessels in the lateral ventricles or IVH without ventricular dilatation (*n* = 8)	IVH without ventricular dilatation or parenchymal hemorrhage (*n* = 7)	*p* value
Risk factors						
Asphyxia						
Hypoxic-ischemic encephalopathy, n (%)	145 (100)	15 (100)	-	8 (100)	7 (100)	-
Brain hypoxic-ischemic injury, n (%)	67 (46)	5 (38)	NS	3 (38)	3 (60^a^)	NS
Treatment						
Hypothermia/rewarming, n (%)	145 (100)	15 (100)	-	8 (100)	7 (100)	^−^
Hemodynamic instability						
Mechanical ventilation, n (%)	98 (68)	10 (67)	NS	4 (50)	5(71)	NS
Lowest pCO2 level	29.2 ± 8.78	28.7 ± 4.2	NS	30.1 ± 3.4	26.8 ± 4.8	NS
Highest pCO2 level	55.4 ± 15.6	57.1 ± 12.7	NS	55.7 ± 13.4	59.0 ± 12.6	NS
Highest pCO2variations	27.0 ± 15.6	28.4 ± 15.1	NS	25.6 ± 15.6	32.2 ± 14.8	NS
Duration of mechanical ventilation, days	3.3 ± 5.0	3.5 ± 4.1	NS	1.4 ± 1.9	5.8 ± 4.8	NS
Persistent pulmonary hypertension, n (%)	46 (32)	6 (40)	NS	2(25)	4(57)	NS
Hypotension, n (%)	81 (56)	10 (67)	NS	5 (63)	5(71)	NS
Hemostasis disturbances						
Thrombocytopenia, n (%)	34 (23)	7(47)	NS	2(25)	5(71)	NS
Coagulopathy, n (%)	46 (32)	11 (73)	0.004	7(88)	4(57)	NS
Metabolic disturbances						
Hypoglycemia, n (%)	16 (11)	3 (20)	NS	1 (13)	2 (29)	NS
Other						
Venous sinus thrombosis, n (%)	3(2)	0(0)	NS	0(0)	0(0)	-
Traumatic birth/instrumental delivery, n (%)	34 (23)	5 (33)	NS	2(25)	3(43)	NS

^a^Two patients did not have a brain MRI or an autopsy to define the extent of brain surgery.

*Abbreviations*: *IVH* intraventricular hemorrhage, *NS* non-significant

When comparing asphyxiated newborns developing IVH to the asphyxiated newborns not developing IVH enrolled in the cohort, coagulopathy was more frequent in these IVH patients (*p = 0.004*). When compared separately, mechanical ventilation, persistent pulmonary hypertension, hypotension, thrombocytopenia, hypoglycemia, and traumatic delivery were not statistically different between the two groups. However, when combining the risk factors together, asphyxiated newborns developing IVH presented significantly more often with a combination of persistent pulmonary hypertension, hypotension, thrombocytopenia, and coagulopathy (*p = 0.04*).

Brain MRIs after hypothermia or autopsy results were obtained in 87 % (13/15) of the asphyxiated newborns developing IVH. Brain injury was present in 46 % (6/13) of them, with a watershed injury pattern in 67 % (4/6) and a total cortical injury pattern in 33 % (2/6). Sixty-one percent (8/13) had subdural hematoma, and 8 % (1/13) had epidural hematomas. None presented evidence of sinus venous thrombosis.

## Discussion

The incidence of IVH was 9 % (95 % CI: 5.3-15.0 %) in our cohort of asphyxiated newborns treated with hypothermia, which is significantly higher than the reported incidence of 3 % in term asymptomatic newborns [1, 3]. Hypothermia and rewarming cause fluctuations of cerebral blood flow [3–5, 17, 18], as well as depressed cardiac function, hypotension, and some disturbances of the coagulation profile [18]. Asphyxia in itself can have similar effects. Another key factor is that asphyxia very often leads to impaired cerebral autoregulation [17]. All these factors may place the asphyxiated newborns treated with hypothermia at an increased risk of developing IVH.

The timing of IVH in term infants has been poorly studied. It has been reported within the first days of life in those with perinatal risk factors, but up to 3–4 weeks postnatally in those with no clear associated risk factor [1–3]. Our results for asphyxiated newborns treated with hypothermia suggest that IVH manifests most often during the later part of hypothermia (30 % diagnosed during the latest segment of hypothermia) or during the rewarming itself (70 % diagnosed after rewarming). This period probably corresponds to the period of the largest fluctuations of cerebral blood flow in these patients [17, 18]. Hyperperfusion has been noted on days 2–3 of life (*i.e.*, during cooling) in the injured brain areas of these newborns [17, 18]. Rewarming is supposed to restore the cerebral blood flow within the normal range, after having been decreased during hypothermia treatment. Peripheral vasodilatation typically occurs during rewarming, which increases the intravascular blood volume and often leads to hypotension [19]. Rewarming also is thought to cause a mismatch between oxygen delivery and consumption [20, 21]. All these factors could explain why the greatest risk of developing IVH in asphyxiated newborns treated with hypothermia occurs during the latest part of hypothermia or during the rewarming phase. To exactly define the timing of IVH in this population, a systematic study with brain imaging (head ultrasound or brain magnetic resonance imaging) on admission and then daily until after the rewarming should be performed. It may help to determine if a brain imaging on admission or during hypothermia may be a wise addition to the standard imaging protocol for these newborns.

The pathogenesis of IVH in term newborns seems largely related to the disturbances of cerebral blood flow [17, 18]. Respiratory distress and mechanical ventilation with fluctuations of carbon dioxide levels can also increase the risk of IVH in term newborns by causing perturbations of the cerebral blood flow. Other factors that might cause abnormalities of cerebral blood flow include variations of arterial blood pressure [22, 23]. In addition, thrombocytopenia is another well-known risk factor [22, 24]. The role of coagulopathy in the pathogenesis of IVH also has been highlighted. In our cohort of asphyxiated newborns treated with hypothermia, perinatal characteristics between both groups (IVH versus no IVH) were the same, suggesting that both groups had a similar degree of initial asphyxia. However, when comparing each known risk factor of IVH between the two groups, coagulopathy played a significant role, and thrombocytopenia tended also to be more frequent in the asphyxiated newborns developing IVH. When comparing the combined risk factors, the association of four risk factors (*i.e.*, mechanical ventilation, hypotension, thrombocytopenia, and coagulopathy) was again significantly more frequent in the asphyxiated newborns developing IVH, confirming that the most hemodynamically unstable asphyxiated newborns (not unexpectedly) are those at the greatest risk for developing IVH. This increased risk was especially true for those newborns developing IVH with ventricular dilatation or parenchymal hemorrhage.

Interestingly, the overall incidence of persistent pulmonary hypertension and hypotension in our population of newborns was higher compared to the available systemic database review of asphyxiated newborns [16]. The incidence of persistent pulmonary hypertension was 15 % in the Cochrane database review [16] compared to 32 % in the newborns in our study not developing IVH and 40 % of those who developed IVH in our study. The incidence of hypotension was 51 % in the Cochrane database review [16] versus 56 and 67 % in our population of newborns. Our incidence of thrombocytopenia, coagulopathy, and hypoglycemia was comparable with that described in the Cochrane database systemic review [16]. The incidence of thrombocytopenia was present in 31 % in the Cochrane database review [16] versus 23 and 47 % in our study. Coagulopathy was present in 31 % in the Cochrane database review [16] versus 32 and 73 % in our study. Hypoglycemia was present in 16 % in the Cochrane database review [16] versus 11 and 20 % in our study. In our population of patients, these different risk factors seemed to slowly improve over time, with a decreased incidence during the rewarming mainly for the group with the IVH limited to the choroid plexus or with IVH without associated documented ventricular dilatation.

Thirty-eight percent of the asphyxiated newborns developing IVH had signs of brain injury, 60 % presented with a watershed injury pattern, and 40 % with a total cortical injury pattern that included both white and cortical gray matter injuries [15]. These parenchymal injuries possibly increase the fragility of brain tissue around the ventricle, which may lead to the hemorrhagic transformation of the tissue in the most susceptible patients. Interestingly, none of the asphyxiated newborns developing IVH presented with a basal ganglia injury pattern. Also, none of these patients presented with obvious sinus venous thrombosis, one of the most commonly reported risk factors for IVH in term newborns in the literature [25].

The main limitation of this study is that it is a cohort study; a randomized control trial would have been more ideal to address whether the incidence of IVH is increased when the newborns are treated with hypothermia. However, as cooling is now the standard of care, it is no longer possible to randomize infants to cooling or not to assess the complications of cooling for neonatal encephalopathy such as IVH. As such, this cohort study is the most feasible means of addressing the question. Also only 15 newborns developed documented intraventricular and/or intraparenchymal hemorrhage - a relatively small number that did not allow us to conduct a multivariate regression analysis due to the limited number of IVH cases. Only studies that could combine several databases of such patients in a systematic way would provide a large enough sample to conclude whether the trends observed in the present study could be confirmed. Another limitation of our study is that it did not have a uniform protocol for repeated imaging (magnetic resonance imaging and/or head ultrasounds) during hypothermia - except for the newborns enrolled in the MRI research study - that was used prospectively to determine the timing of IVH. It may be worthwhile to develop such a protocol to determine whether the incidence of IVH was underestimated in our study. This protocol should include a gradient echo (GRE) T2*-weighted sequence or a susceptibility-weighted imaging (SWI) sequence to ensure the adequate visualization of the IVH. However, despite these limitations, the results presented here remain of interest, since the major randomized controlled studies about hypothermia in asphyxiated newborns [10–13] did not address this issue, and, if in truth a threefold increase in IVH risk exists for infants undergoing hypothermia, this risk could impact management, especially regarding the maintenance of hemodynamic stability and rewarming.

## Conclusions

Asphyxiated newborns treated with hypothermia may be at an increased risk of intraventricular hemorrhage, especially those with significant hemodynamic instability. Intraventricular hemorrhage seems to develop most often during the latest part of hypothermia treatment or during the rewarming. Thus, efforts should be directed towards maintaining hemodynamic stability in these patients, even during the rewarming phase.

## Abbreviation

IVH: Intraventricular hemorrhage
